# Spatial and temporal distribution of foot and mouth disease outbreaks in Amhara region of Ethiopia in the period 1999 to 2016

**DOI:** 10.1186/s12917-020-02411-6

**Published:** 2020-06-09

**Authors:** Endris Aman, Wassie Molla, Zeleke Gebreegizabher, Wudu Temesgen Jemberu

**Affiliations:** 1 College of Agriculture and Environmental Science, Bahir Dar University, P.O. BOX 79, Bahir Dar, Ethiopia; 2 College of Veterinary Medicine and Animal sciences, University of Gondar, P.O.BOX 196, Gondar, Ethiopia; 3 Livestock Development Agency, Amhara National Regional State, P.O. BOX 437, Bahir Dar, Ethiopia

**Keywords:** Amhara region, Ethiopia, FMD, Outbreak, Spatial, Temporal

## Abstract

**Background:**

Foot and mouth disease (FMD) is an economically important trans-boundary viral disease of cloven-hoofed animals. It is caused by FMD virus, which belongs to the genus *Aphthovirus* and family *Picornaviridae*. FMD is a well-established endemic disease in Ethiopia since it was first detected in 1957. This retrospective study was carried out to identify the spatial and temporal distribution of FMD outbreaks in Amhara region of Ethiopia using 18 years (January 1999–December 2016) reported outbreak data.

**Results:**

A total of 636 FMD outbreaks were reported in Amhara region of Ethiopia between 1999 and 2016 with an average and median of 35 and 13 outbreaks per year respectively. In this period, FMD was reported at least once in 58.5% of the districts (n = 79) and in all administrative zones of the region (n = 10). The average district level incidence of FMD outbreaks was 4.68 per 18 years (0.26 per district year). It recurs in a district as epidemic, on average in 5.86 years period. The incidence differed between administrative zones, being the lowest in East Gojjam and highest in North Shewa. The occurrence of FMD outbreaks was found to be seasonal with peak outbreaks in March and a low in August. The long-term trend of FMD outbreaks indicates a slight, but statistically significant (*p* < 0.001) decrease over the study period.

**Conclusion:**

FMD occurred in all zones of the region and showed statistically significant decrease in the long-term trend. Numbers of outbreaks were relatively higher during dry season. The spatial and temporal distribution identified in this study should be considered in controlling the disease. As unregulated and frequent animal movements are the likely causes of high outbreak occurrence during the dry season, animal movement regulations should be considered for the long-term control of FMD.

## Background

Foot and mouth disease (FMD), is an economically important trans-boundary viral disease of cloven-hoofed animals. Foot and mouth disease virus (FMDV), the cause of the disease, belongs to the genus *Aphthovirus* and family *Picornaviridae*. The virus affects a wide range of hosts including domestic and wild ruminants and pigs. According to OIE (World Organization for Animal Health), FMD was the first viral infection of animals recognised and ranks first among the diseases of animals [1]. FMD is one of the most important livestock diseases responsible for the loss of production and productivity, trade embargoes and huge control costs across the globe [2].

FMDV has seven distinct serotypes: A, O, C, and South African Territories (SAT) 1, SAT 2, SAT 3, and Asia 1. Each serotype has many biotypical strains and topotypes, with incomplete cross protection [3, 4]. Of these seven serotypes, serotype O and A have the broadest distribution in Africa [5, 6]. The occurrence of FMD due to serotype C seems decreasing in recent time. Serotype C occurrence was reported for the last time in Kenya in 2004 and in Ethiopia in 2008 [7, 8].

In Ethiopia, FMD is a well-established endemic disease since its detection in 1957 for the first time [9, 10]. Previous studies in the country reported FMD in different animal species with different prevalence levels. For example, in cattle they reported FMD prevalence that ranges from 1.4 to 53.6% at animal level and up to 61% at herd level [11–13], in domestic small ruminants 4 to 11%, and in ungulate wildlife 30% [14, 15]. Among the known FMD serotypes, four serotypes (A, O, SAT 1 and SAT 2) are maintained endemically in Ethiopia. Published articles on FMD show that type A and O are the main serotypes responsible for significant economic losses in the country [6, 8, 16].

In recent times, there is an interest to control FMD in Ethiopia to boost its live animal and meat exports as the disease is considered the main obstacle to the international trade of livestock and their products [17]. However, controlling of FMD in endemic developing countries in general and in Ethiopia in particular is not an easy task due to several reasons. These include the wide distribution of multiple serotypes and subtypes, and the presence of diverse hosts of the virus across the country, uncontrolled livestock movement and shortage of effective and affordable FMD vaccines in sufficient quantities in the country. Besides, FAO and OIE advocate that the control of FMD in endemic countries like Ethiopia has to be carried out in a long-term progressive risk reduction approach [18, 19].

Currently, in Ethiopia, there is planning towards a national FMD control program, which is a part of progressive control pathway (PCP). Ethiopia has been identified to be in stage one of the FMD progressive control pathway. The progressive control path way requires detail epidemiological studies to locate and identify the spatiotemporal distribution of FMD in the country to progress to the next stage. In spite of a number of sero-prevalence studies conducted in different regions of Ethiopia, there are no studies that investigated the spatial and temporal distribution of FMD, particularly in Amhara region. Therefore, this study was undertaken with the objective of identifying the spatial and temporal distribution of the reported outbreaks of FMD from January 1999 to December 2016 in Amhara region of Ethiopia.

## Results

### Incidence of FMD outbreaks

A total of 636 FMD outbreaks were reported in Amhara region of Ethiopia between 1999 and 2016 with an average and median of 35 and 13 outbreaks per year respectively. In this period, FMD was reported at least once in 58.5% of the districts (n = 79) and in all administrative zones of the region (n = 10). Majority of the outbreaks were from North Shewa zone (44%), South Wollo zone (18.5%), West Gojjam zone (11.16%) and Awi zone (7%) (Additional file 1: Fig. S1). The average incidence of FMD outbreaks at district level was 4.68/18 district years or 0.26/ district year.

The reoccurrence of FMD outbreak in the districts of Amhara region varies from 1 to 17 years. The average time for the reoccurrence of the disease in the same district is 5.86 years. The time between outbreaks was shorter in districts of West Gojjam, Oromia, North Wollo and Awi zones and a bit longer in districts of other six zones.

### Geographical distribution of FMD outbreaks

**Fig. 1:**
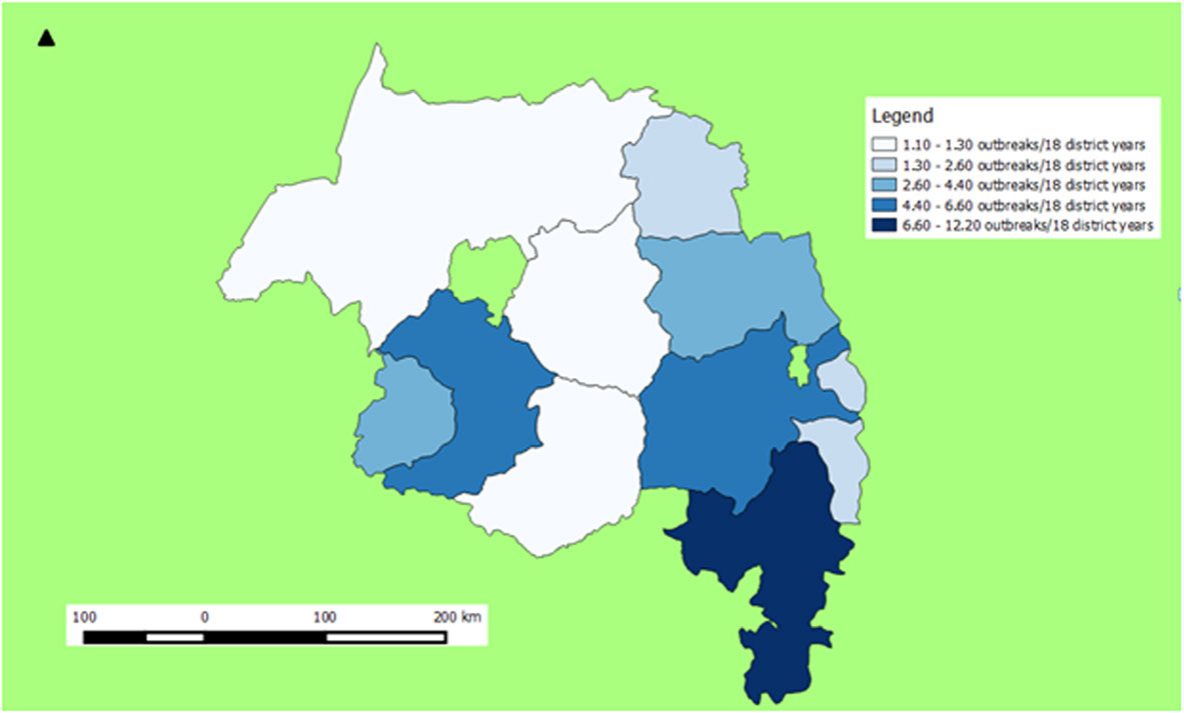
Zonal distribution of FMD outbreaks per 18 district years in Amhara region over the period 1999–2016 (we created the map by QGIS version 3.10.2 (a Coruna) software (https://qgis.org/en/site/forusers/download.html))

The geographical distribution of the 18 years FMD outbreaks within the Amhara region is depicted in Fig. 1. FMD has been reported from all zones (*n* = 10) of Amhara region during the period 1999–2016. The outbreak incidence mapped by the administrative zones showed that the eastern part of the region was more affected by FMD outbreak than the western part (Fig. 1). The FMD outbreak incidence was above the average incidence of the region in North Shewa (12.2/18 district years), South Wollo (6.6/18 district years) and West Gojjam (5.5/18 district years).

### Temporal trends of FMD outbreak occurrences in Amhara region

**Fig. 2:**
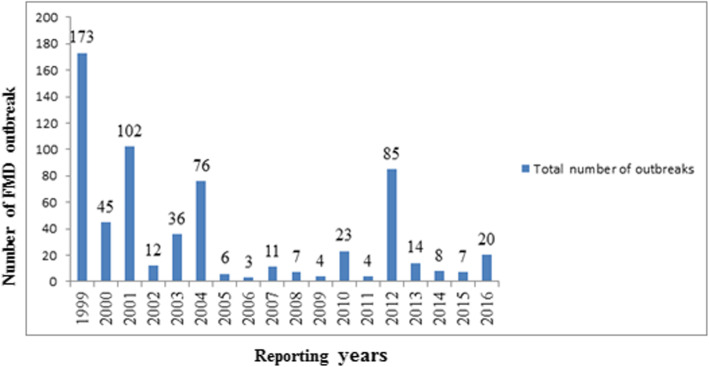
Annual FMD outbreaks in Amhara region from 1999 to 2016

**Fig. 3:**
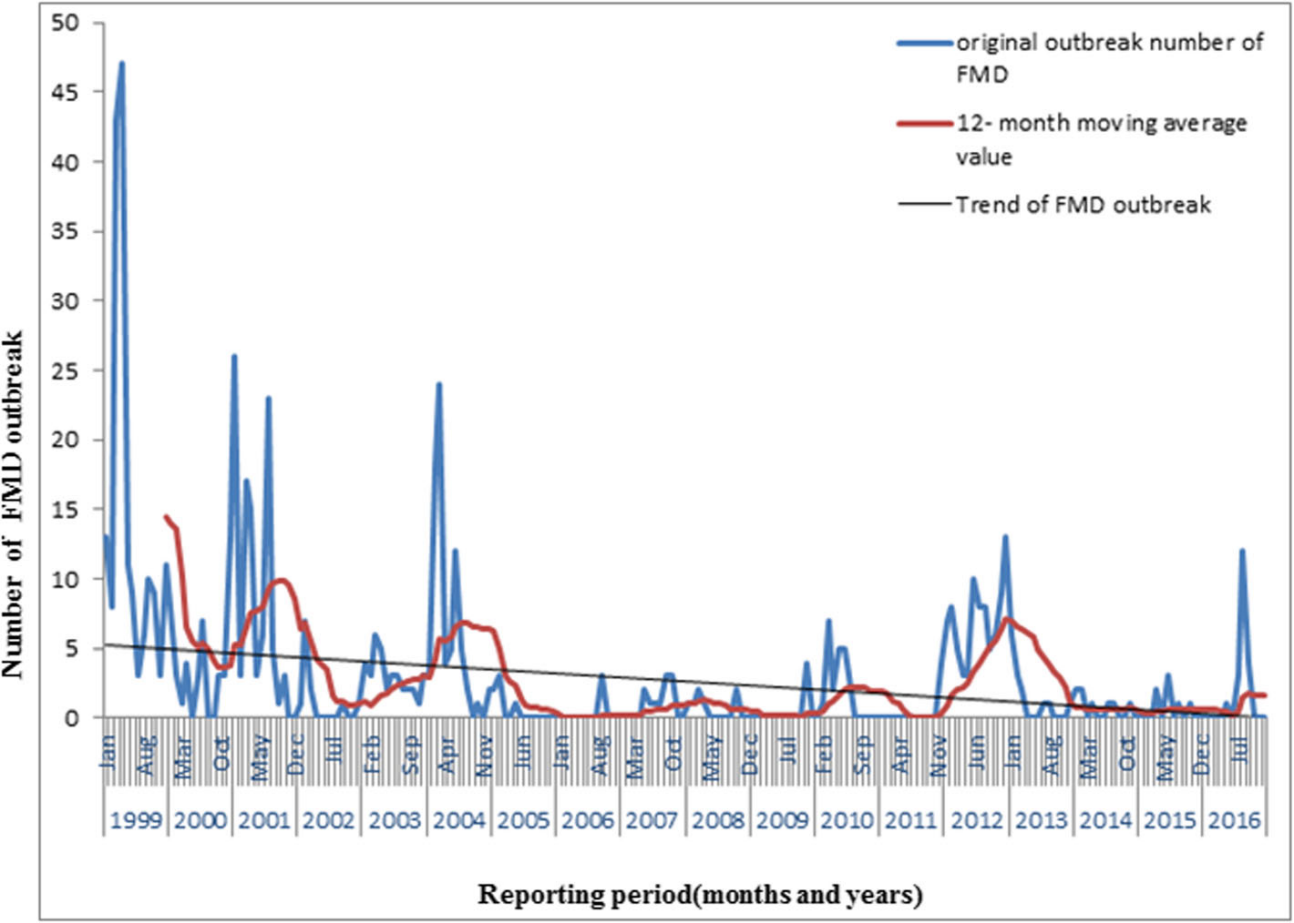
Monthly outbreak and trend of FMD outbreak from 1999 to 2016

**Fig. 4:**
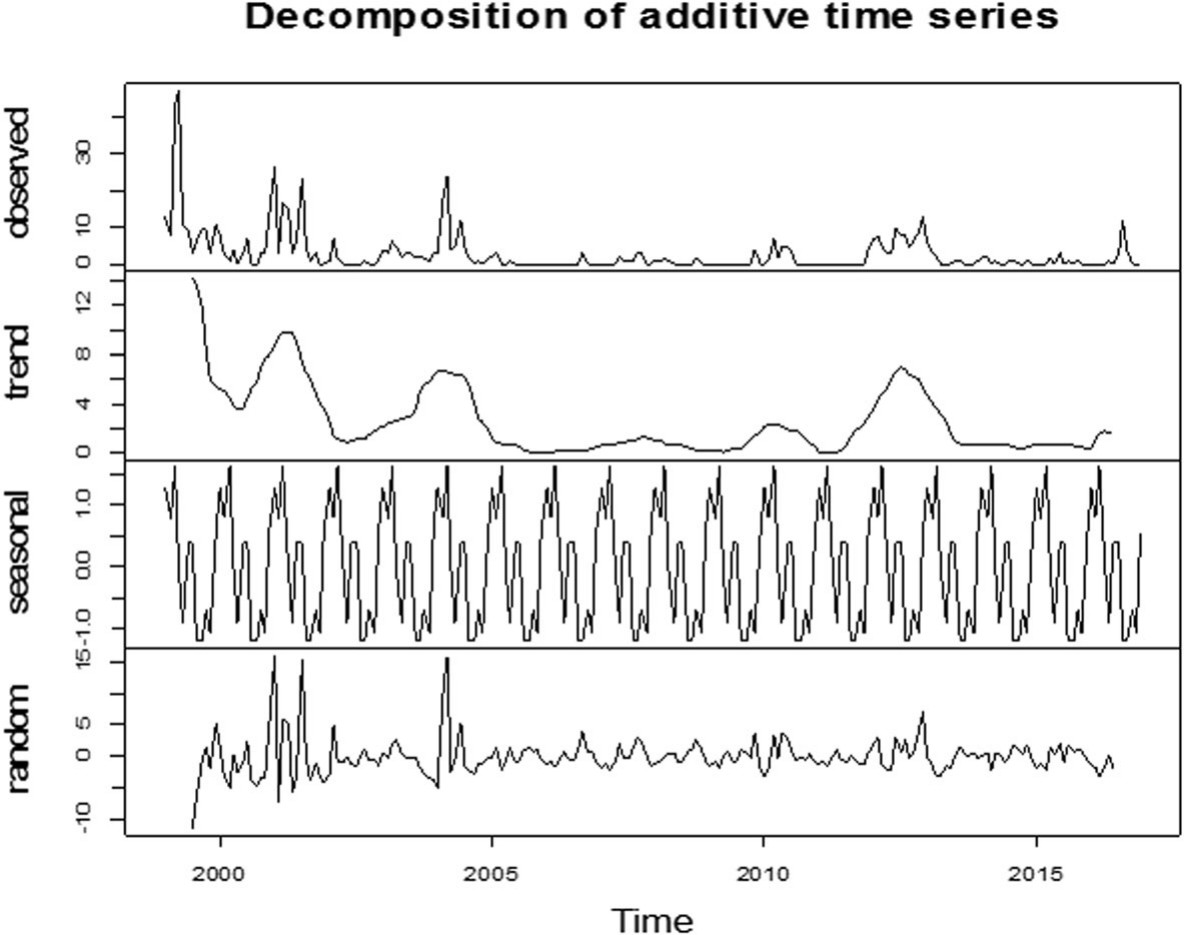
Decomposition of the time series of the observed temporal pattern of FMD outbreaks (top panel) into three components: trend, seasonality and random

**Fig. 5:**
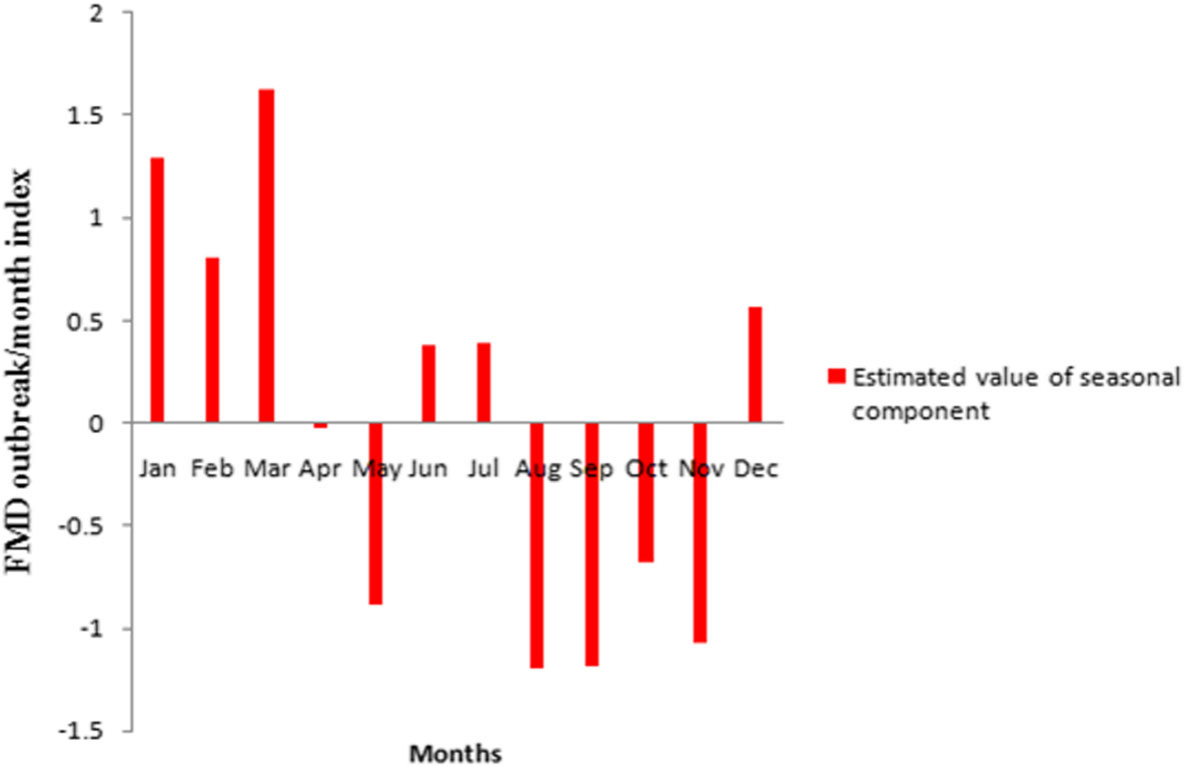
Seasonal indices of monthly FMD outbreak between 1999 and 2016 in Amhara region

In the period of January 1999 to December 2016, high number of FMD outbreaks were reported in 1999 (*n* = 173 outbreaks), 2001 (*n* = 102), 2004 (*n* = 76) and 2012 (*n* = 85) while the lowest number of outbreaks were reported in 2006 (*n* = 3) (Fig. 2, Additional file 2: Table S1and Additional file 3: Table S2). The highest number of outbreaks were reported in the month of March (*n* = 109 across all years), which accounted for 17.14% of all reported outbreaks and the lowest in November (*n* = 21), accounting for 3.3% of all reported outbreaks (Additional file 2: Table S1). The monthly distribution of FMD outbreaks is presented in Fig. 3 and Additional file 2: Table S1. The decomposed FMD outbreak occurrence pattern shows a slight decrease in the number of monthly outbreaks (Fig. 4), which was statistically significant (P < 0.001). The seasonality in the numbers of outbreaks was apparent as it can be seen in Figs. 3 and 4. Runs test for test of randomness indicates that the temporal distribution of FMD outbreaks was not random. Moreover, plotting of monthly indices of number of outbreaks shows higher frequency of outbreaks in the dry season months compared to other seasons, which demonstrates statistically significant seasonality of FMD outbreaks (Fig. 5). The largest incidence was reported for March (about 1.63) and the lowest for August (about − 1.2), indicating that the number of FMD outbreaks peaked in March and became low in August (Fig. 5 and Additional file 4: Fig. S2). Generally, the number of FMD outbreaks was far above average from December to March and far below average from August to November (Fig. 5).

The trend, seasonal and random components were estimated by decomposing the FMD outbreak time series (Fig. 4). From the trend component, FMD outbreak occurrence appears to have a cycle with a periodicity of two–six years with peaks in 1999, 2001, 2004, 2010 and 2012) (Fig. 4).

## Discussion

Unlike several previous FMD studies in the Amhara region, which were cross-sectional sero-prevalence studies, this is the first longitudinal study to report the spatial and temporal distribution of FMD outbreaks in the region. This study indicated that frequent FMD outbreaks occurred in Amhara region every year with 636 total outbreaks in 18 years. During this period, on average 35 FMD outbreaks were reported annually across the region demonstrating that FMD is endemic in the region. The highest outbreaks were reported in 1999 (n = 173) which is in agreement with Asfaw and Sintaro [20] who reported the highest number of FMD outbreaks in 1999 nationally.

Foot and mouth disease outbreaks were reported from all zones of Amhara region during the study period, with the highest outbreak incidence of up to 280 outbreaks in the North Shewa zone, which is consistent with Ayelet et al. [10] who had reported the highest outbreak in North Shewa zones of both Amhara and Oromia regions. Another report by Jemberu et al. [6] showed the frequent occurrence of FMD outbreaks in the central (which include North Shewa) part of the country, which agrees more or less with the present findings. The high incidence in North Shewa might be due to animal movement from north and north-eastern parts of the country through North Shewa to the central market located in and around Addis Ababa.

The present study documented that about 58.5% of the districts in the region reported at least one FMD outbreak in 18 years, which is lower than the 73% reported by Jemberu et al. [6]. The reason for the discrepancy between the current and the previous study could be due to the difference in the length of the study period and data sources. The previous study was based on short and recent period outbreak history collected from the districts while the present study was based on long period and based solely on official outbreak reports, which might be affected by underreporting.

This study findings indicated that the occurrence of FMD at district level is sporadic, which is in agreement with Belayneh et al. [21], who had reported the epidemic nature of FMD in selected districts of Amhara region. However, endemicity of FMD is maintained in the region, as the outbreaks in different districts of the region do not occur at the same time. The average time for the reoccurrence of the disease in the same districts was 5.86 years. The reoccurrence period of FMD varies across the study districts. Some districts reported outbreaks after 1 year of quiescence, whereas others reported an outbreak of FMD after a longer period (up to 17 years) of quiescence. The difference in the reoccurrence period between districts could be due to the difference in the degree of animal movement, level of geographical isolation by natural physical barriers, and level of herd immunity.

Generally, in Amhara region, the number of FMD outbreaks significantly increase in two to 6 years cycle. This finding is consistent with research reported by Belayneh et al. [21] who concluded that increased number of FMD epidemics occur in Amhara region on average every 2 years. The epidemic cycle indicated in our study is also in line with the epidemic cycle range of three to 6 years reported by previous studies from other endemic countries [22–25].

Temporal patterns of disease outbreaks were decomposed into long-term (secular), cyclical and seasonal trends. The decomposed temporal patterns demonstrated statistically significant seasonality for FMD outbreaks, which is in line with Nejash’s [26] findings who pointed out season as risk factors for FMD outbreak. In contrast to our result, Jemberu et al. [6] reported that FMD outbreak incidence has neither long-term nor seasonal trend in Ethiopia. In the present study, the peak FMD outbreaks were recorded in March (the dry season) and the low in August (the rainy season). The variation of FMD outbreak in season might be related to the variation in animal movements. Outbreak numbers increased during December, January, February and March (reaching peak in March). This might be coinciding with the increased animal transports due to the increased meat demand during the impending Christmas (at the end of December) and Easter festival (March–April) celebrations of the Christian population in the Amhara region, which are the two big religious festivals for Orthodox Christians in Amhara region in particular and in Ethiopia in general. Similarly, peak FMD outbreaks coinciding or following special holidays have been observed in endemic countries such as Sri Lanka. This has been related to animal transports in response to the increased demand of meat for the impending Milad-Un-Nabi (March) and Ramadan festival of the Muslim population as well as the ‘cattle salvage’ practice associated with the two Buddhist festivals Vesak (May) and Poson (June) celebrations [27]. On the other hand, in most midland and highland parts of Amhara region, during the rainy season of the years wide areas of farmland are planted with crops, as a result the movement of domestic animals is restricted and kept confined on small plots of grazing lands, which could be the reason for low incidence of FMD outbreaks in this season.

The trend of FMD outbreaks from January 1999 to December 2016 indicates a slight but statistically significant decrease over the period**.** The observed decrease in FMD outbreak occurrence in Amhara region in the recent years could be due to the effort made to ban unrestricted livestock grazing in the region that resulted in decreased free movement of animals. The possibility of decreasing outbreak reporting rate by the districts cannot also be ruled out. Therefore, it requires further study to conclude that the long-term trend of the disease is decreasing and to know the reason of the decreasing trend.

The results of this study might possibly be biased by the outbreak reporting rate of the districts. Jemberu et al. [6] have already documented a serious underreporting of FMD outbreaks in Ethiopia. The impact of underreporting will have more bias, if the underreporting is different across districts and time. In addition, the reported outbreaks are mostly diagnosed based on clinical signs without confirmatory diagnostic tests, which may impact the accuracy of the reported outbreak incidences. Even though our study might have the above mentioned limitations, it generally attempted to generate quite important epidemiological information about the spatial and temporal distribution of FMD in the study area, which could be valuable inputs to support the regional and national decision making regarding FMD control.

## Conclusion

FMD is wide spread and well established in Amhara region. It occurred in all zones and more than half of the districts in the region experienced at least one FMD outbreak in the time between 1999 and 2016. Outbreaks are seasonal and occurred more often in the dry season months. Increased number of outbreaks occur in the region with epidemic cycle of two to 6 years. Identification of temporal patterns and spatial distribution can indicate particular times and areas when and where attention should be given to control and prevent the disease in cost effective ways. Hence, strategic prophylactic vaccination of animals should commence at the beginning of the dry season together with restrictions on animal movements during dry season months and farmers of the Amhara region have to be aware about the risk of unrestricted animal movement. Since the current study covers only the Amhara region, subsequent study needs to be undertaken in Ethiopia in general to have a good insight in the spatial and temporal distribution of FMD at national level.

## Methods

### Study area

**Fig. 6:**
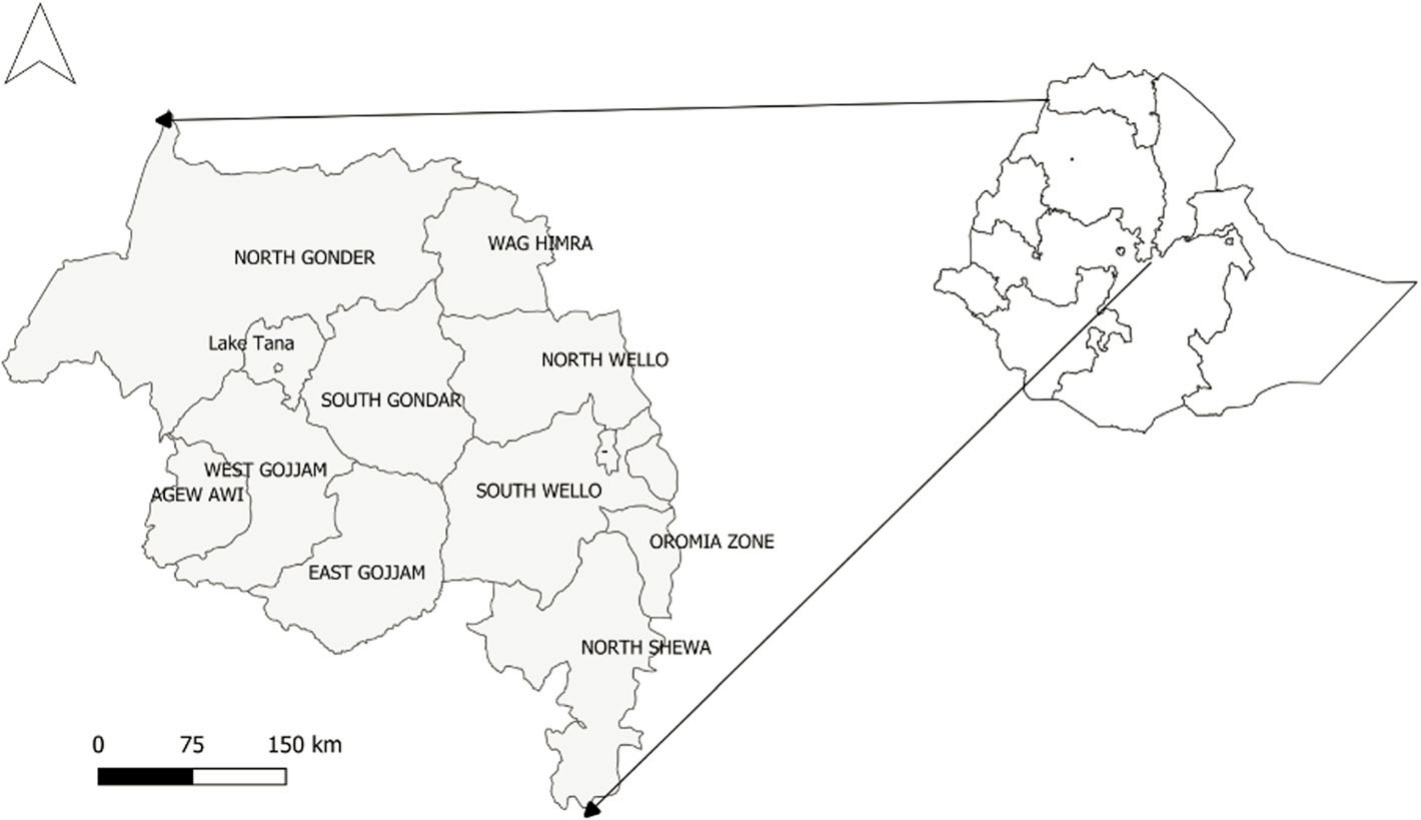
Map of Ethiopia showing the study area Amhara region with administrative zone divisions (we created the map by QGIS version 3.10.2 (a Coruna) software (https://qgis.org/en/site/forusers/download.html))

The study was conducted in Amhara region of Ethiopia (Fig. 6). The Amhara region is located in the north western part of Ethiopia between 9°20′ and 14°20′ North latitude and 36° 20′ and 40° 20′ East longitude. The region covers approximately 161,828.4 km^2^ area and is moderately compact in shape. It consists of three major agro-ecological zones. These are highlands (above 2300 m above sea level (masl)), mid-highlands (1500 to 2300 masl) and lowlands (below 1500 masl) accounting for 20, 44 and 28% of the land area respectively. A little over 50% of the total area of the region is considered potentially arable for agricultural production activities [28]. The daily temperature ranges from 16 to 27 °C. The mean annual rainfall over the whole region varies from 300 mm in the East to well over 2000 mm in the West [28].

The region is made up of 11 administrative zones, namely Wag Hemra, North Wollo, North Gondar, South Gondar, South Wollo, North Shewa, Oromia, East Gojjam, West Gojjam, Awi and Bahir Dar special zone. These administrative zones are divided into a total of 136 districts and 3429 kebeles [28]. Based on the 2007 census of the central statistical agency of Ethiopia (CSA), the Amhara region has a population of 17,221,976 (8,641,580 men and 8,580,396 women) [29]. The 2016/17 CSA report estimated that farmers in Amhara region had a total of 15.98 million cattle (representing 26.8% of Ethiopia’s total cattle population), 9.79 million sheep (representing 31.8% of Ethiopia’s total sheep population) and 6.08 million goats (representing 20.1% of Ethiopia’s total goat population) [30].

### Outbreak data source

FMD is among OIE listed viral diseases, which requires reporting of all occurrences to the national veterinary authority of each OIE member country and internationally to the World Organization for Animal Health. FMD outbreak data were obtained from Bahir Dar and Kombolcha regional veterinary laboratories of Amhara region and the Federal Veterinary Epidemiology Directorate of Ethiopia for the period 1999–2016. The regional and the federal outbreak records were combined to increase the sensitivity of detecting the FMD outbreaks in the region. The records included information such as location, species affected, index date, number of cases, number of outbreaks, number of deaths, and number of animals at risk. For this study, an outbreak was defined as one or more cattle, sheep or goats showing FMD signs in a district. Therefore, the FMD outbreak incidence was computed at district (n = 136) level using the 18 years (January 1999 –December 2016) outbreak data. As stated in the study area, Amhara region has 11 administrative zones, however, Bahir Dar became a special zone recently (previously it was under West Gojjam zone) so the outbreaks reported from Bahir Dar district were included under West Gojjam zone.

### Data analysis

Microsoft Excel spreadsheets (Microsoft Corporation) were used to manage the data and draw graphs. Descriptive methods were used to calculate outbreak incidence. The mean FMD outbreak incidence was calculated by summing all reported FMD outbreaks over the study period in the region divided by the total number of districts and number of years (district years). The spatial distribution of FMD outbreaks over the study period was drawn by administrative zones using QGIS version 3.10.2 software.

The number of FMD outbreaks reported in the 18 year study period were graphed to visualize the temporal trends of the disease. The graph was inspected for the presence of seasonality or long-term trend. The long-term trend of FMD outbreaks was verified by linear regression in STATA version 14 by taking the number of FMD outbreaks as outcome variable and years of the outbreaks as predictor variable. Identification and estimation of the three components of the temporal additive model: long-term trend, seasonality, and irregularity was performed by decomposing the FMD outbreak time series with package ‘TTR’ (Technical Trading Rules) in R software.

To assess the seasonality of FMD outbreaks, 12 months moving averages were calculated and, plotted using the outbreak numbers over the 18 years study period. The moving averages were used to reduce random variation and to ease the detection of underlying trends [31]. Finally, the run test of randomness was used to verify the randomness pattern of the monthly occurrence of FMD outbreaks [32].

## Supplementary information

**Additional file 1 Figure S1.** Distribution of FMD outbreaks (*n* = 636) over administrative zones of Amhara region in the period 1999–2016.

**Additional file 2 Table S1.** Number of FMD outbreaks reported monthly over the period 1999–2016 in Amhara region.

**Additional file 3 Table S2.** Number of FMD outbreaks reported yearly in each zone of Amhara region over the period 1999–2016.

**Additional file 4 Figure S2.** Seasonality of FMD outbreak in Amhara region using estimated seasonal value.

## Data Availability

The data on which our findings are based included in this manuscript and its additional information files.

## Abbreviations

CSA: Central statistical agency
FAO: Food and agriculture organization
FMD: Foot and mouth disease
GIS: Geographical Information System
Masl: meter above sea level
OIE: Office International des Epizooties (World organization for animal health)
PCP: Progressive control pathway
QGIS: Quantum Geographical Information System
SAT: South African Territories
TTR: Technical Trading Rules
