# Supplementary material for: Spatial and temporal distribution of foot and mouth disease outbreaks in Amhara region of Ethiopia in the period 1999 to 2016
Source: BMC Vet Res. 2020 Jun 9;16:185. doi: 10.1186/s12917-020-02411-6 (PMC7285603; doi:10.1186/s12917-020-02411-6)
Supplement: Supplementary file 2 — Additional file 2 Table S1. Number of FMD outbreaks reported monthly over the period 1999–2016 in Amhara region. [file 12917_2020_2411_MOESM2_ESM.docx]

| Year | Jan | Feb | Mar | Apr | May | Jun | Jul | Aug | Sep | Oct | Nov | Dec | Year total |
| --- | --- | --- | --- | --- | --- | --- | --- | --- | --- | --- | --- | --- | --- |
| 1999 | 13 | 8 | 43 | 47 | 11 | 9 | 3 | 6 | 10 | 9 | 3 | 11 | **173** |
| 2000 | 8 | 3 | 1 | 4 | 0 | 3 | 7 | 0 | 0 | 3 | 3 | 13 | **45** |
| 2001 | 26 | 3 | 17 | 15 | 3 | 6 | 23 | 5 | 1 | 0 | 0 | 1 | **102** |
| 2002 | 1 | 7 | 2 | 0 | 0 | 0 | 0 | 0 | 1 | 0 | 0 | 1 | **12** |
| 2003 | 4 | 3 | 6 | 5 | 2 | 3 | 3 | 2 | 2 | 2 | 1 | 3 | **36** |
| 2004 | 3 | 18 | 24 | 4 | 5 | 12 | 5 | 2 | 0 | 1 | 0 | 2 | **76** |
| 2005 | 2 | 3 | 0 | 0 | 1 | 0 | 0 | 0 | 0 | 0 | 0 | 0 | **6** |
| 2006 | 0 | 0 | 0 | 0 | 0 | 0 | 0 | 0 | 3 | 0 | 0 | 0 | **3** |
| 2007 | 0 | 0 | 0 | 0 | 2 | 1 | 1 | 1 | 3 | 3 | 0 | 0 | **11** |
| 2008 | 1 | 1 | 2 | 1 | 0 | 0 | 0 | 0 | 0 | 2 | 0 | 0 | **7** |
| 2009 | 0 | 0 | 0 | 0 | 0 | 0 | 0 | 0 | 0 | 0 | 4 | 0 | **4** |
| 2010 | 0 | 1 | 7 | 2 | 5 | 5 | 3 | 0 | 0 | 0 | 0 | 0 | **23** |
| 2011 | 0 | 0 | 0 | 0 | 0 | 0 | 0 | 0 | 0 | 0 | 0 | 4 | **4** |
| 2012 | 7 | 8 | 5 | 3 | 3 | 10 | 8 | 8 | 5 | 6 | 9 | 13 | **85** |
| 2013 | 6 | 3 | 2 | 0 | 0 | 0 | 1 | 1 | 0 | 0 | 0 | 1 | **14** |
| 2014 | 2 | 2 | 0 | 1 | 0 | 0 | 1 | 1 | 0 | 0 | 1 | 0 | **8** |
| 2015 | 0 | 0 | 0 | 2 | 0 | 3 | 0 | 1 | 0 | 1 | 0 | 0 | **7** |
| 2016 | 0 | 0 | 0 | 0 | 1 | 0 | 3 | 12 | 4 | 0 | 0 | 0 | **20** |
| **Total** | **73** | **60** | **109** | **84** | **33** | **52** | **58** | **39** | **29** | **30** | **21** | **48** | **636** |
