# Supplementary material for: Spatial and temporal distribution of foot and mouth disease outbreaks in Amhara region of Ethiopia in the period 1999 to 2016
Source: BMC Vet Res. 2020 Jun 9;16:185. doi: 10.1186/s12917-020-02411-6 (PMC7285603; doi:10.1186/s12917-020-02411-6)
Supplement: Supplementary file 3 — Additional file 3 Table S2. Number of FMD outbreaks reported yearly in each zone of Amhara region over the period 1999–2016. [file 12917_2020_2411_MOESM3_ESM.docx]

| Zones | 1999 | 2000 | 2001  0  1 | | 2002 | 2003 | 2004 | 2005 | 2006 | 2007 | 2008 | 2009 | 2010 | 2011 | 2012  1  2 | 2013  1  3 | 2014  1  4 | 2015  1  5 | 2016  6 | **Total**  **a**  **l** |
| --- | --- | --- | --- | --- | --- | --- | --- | --- | --- | --- | --- | --- | --- | --- | --- | --- | --- | --- | --- | --- |
| Awi | 0 | 0 | | 2 | 0 | 2 | 0 | 0 | 0 | 3 | 3 | 0 | 22 | 2 | 8 | 1 | 0 | 0 | 1 | **44** |
| East Gojjam | 7 | 0 | | 1 | 0 | 1 | 3 | 0 | 0 | 1 | 3 | 0 | 0 | 0 | 1 | 0 | 0 | 0 | 2 | **19** |
| North Gondar | 0 | 0 | | 1 | 7 | 1 | 0 | 0 | 0 | 3 | 1 | 0 | 0 | 0 | 7 | 0 | 0 | 0 | 0 | **20** |
| North Shewa | 93 | 4 | | 38 | 1 | 16 | 64 | 1 | 0 | 0 | 0 | 4 | 1 | 2 | 29 | 5 | 1 | 6 | 15 | **280** |
| North Wollo | 4 | 0 | | 15 | 0 | 2 | 3 | 1 | 3 | 2 | 0 | 0 | 0 | 0 | 4 | 1 | 0 | 0 | 0 | **35** |
| Oromia | 7 | 2 | | 2 | 0 | 1 | 3 | 1 | 0 | 0 | 0 | 0 | 0 | 0 | 2 | 0 | 0 | 0 | 0 | **18** |
| South Gondar | 6 | 0 | | 3 | 1 | 1 | 0 | 0 | 0 | 0 | 0 | 0 | 0 | 0 | 3 | 0 | 0 | 0 | 0 | **14** |
| South Wollo | 47 | 17 | | 0 | 0 | 7 | 3 | 3 | 0 | 0 | 0 | 0 | 0 | 0 | 26 | 7 | 7 | 1 | 0 | **118** |
| Waghemra | 9 | 0 | | 2 | 0 | 1 | 0 | 0 | 0 | 0 | 0 | 0 | 0 | 0 | 5 | 0 | 0 | 0 | 0 | **17** |
| West Gojjam | 0 | 22 | | 38 | 3 | 4 | 0 | 0 | 0 | 2 | 0 | 0 | 0 | 0 | 0 | 0 | 0 | 0 | 2 | **71** |
| **Total** | **173** | **45** | | **102** | **12** | **36** | **76** | **6** | **3** | **11** | **7** | **4** | **23** | **4** | **85** | **14** | **8** | **7** | **20** | **636** |
