# Supplementary material for: Spatial and temporal distribution of foot and mouth disease outbreaks in Amhara region of Ethiopia in the period 1999 to 2016
Source: BMC Vet Res. 2020 Jun 9;16:185. doi: 10.1186/s12917-020-02411-6 (PMC7285603; doi:10.1186/s12917-020-02411-6)
Supplement: Supplementary file 3 [file 12917_2020_2411_MOESM1_ESM.docx]

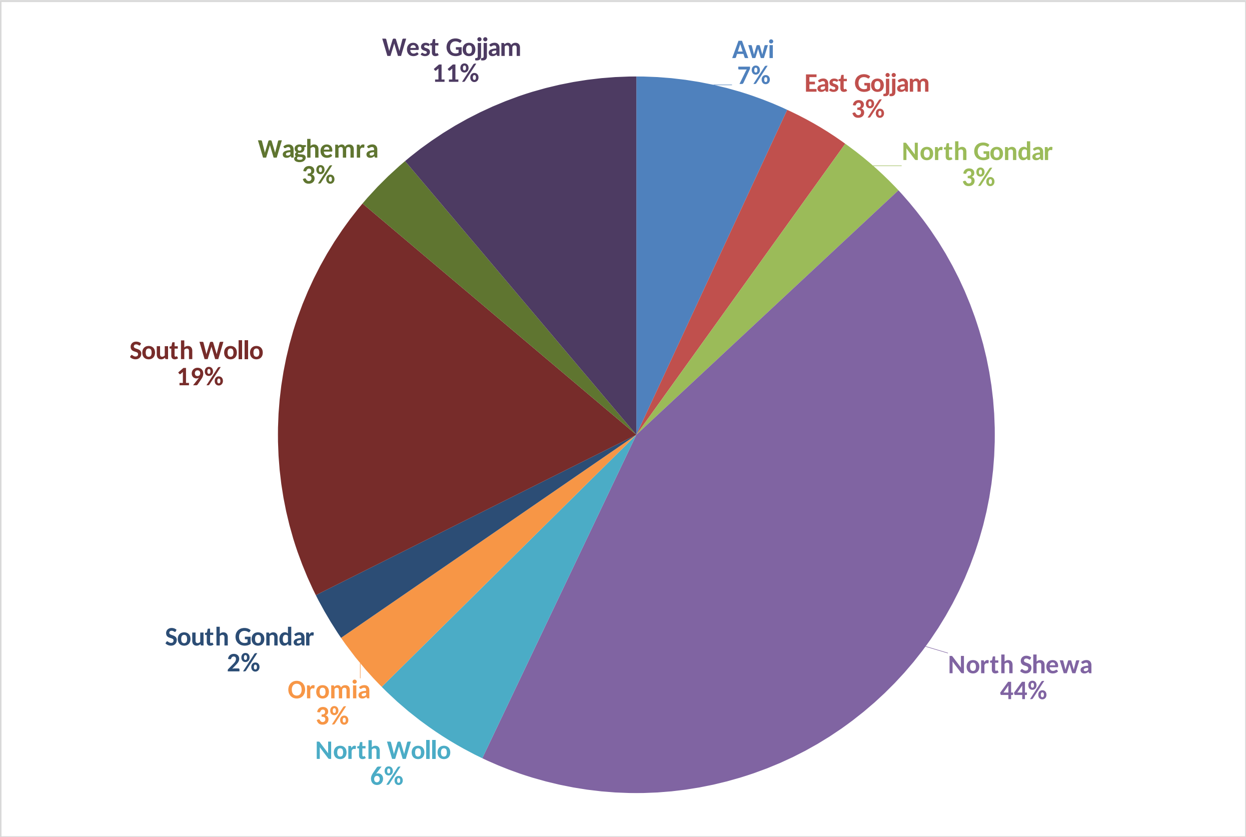
West Gojjam
11%
Awi
7%
East Gojjam
3%
North Gondar
3%
Waghemra
3%
South Wollo
19%
South Gondar
2%
Oromia
3%
North Wollo
6%
North Shewa
44%
