# Supplementary material for: Spatial and temporal distribution of foot and mouth disease outbreaks in Amhara region of Ethiopia in the period 1999 to 2016
Source: BMC Vet Res. 2020 Jun 9;16:185. doi: 10.1186/s12917-020-02411-6 (PMC7285603; doi:10.1186/s12917-020-02411-6)
Supplement: Supplementary file 4 [file 12917_2020_2411_MOESM4_ESM.docx]

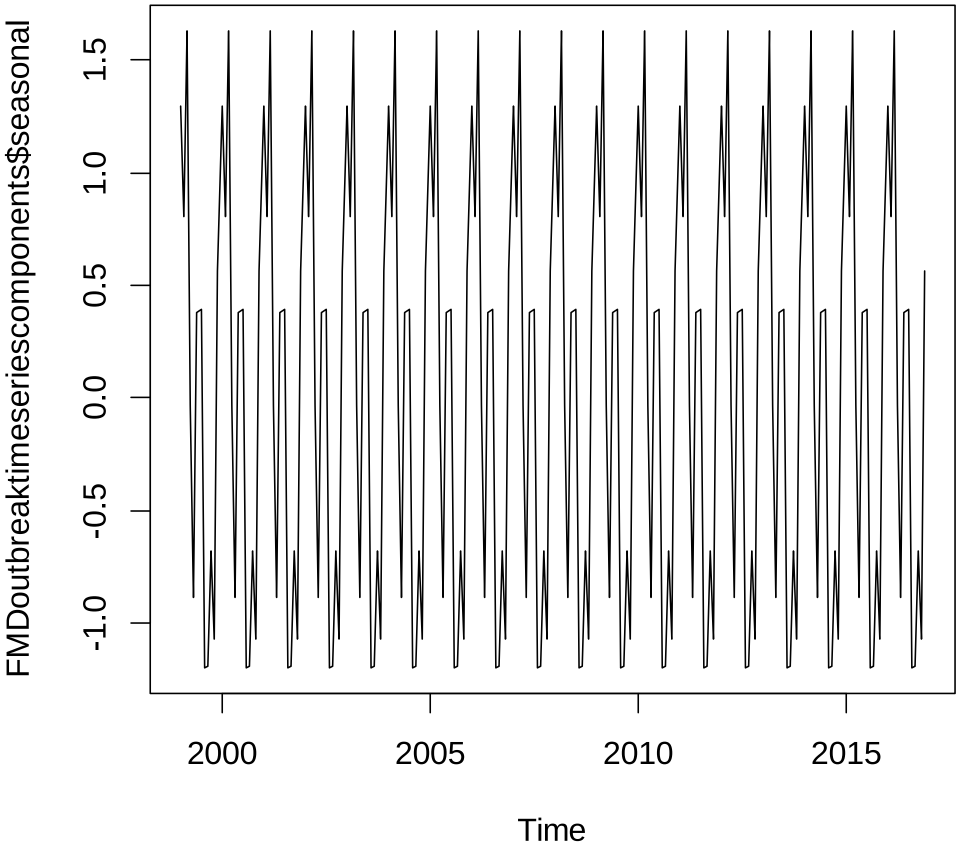
FMDoutbreaktimeseriescomponents$seasonal
-1.0
-0.5
0.0
0.5
1.0
1.5
2000
2005
2010
2015
Time
